# Effect of Lubricant Use on Cervicovaginal Cytology – What's the Evidence?

**DOI:** 10.1055/s-0043-1776025

**Published:** 2023-11-29

**Authors:** Diana Magalhães dos Santos, Maria Beatriz Freire Oliveira Coelho de Matos, Ana Raquel Borges Sousa, Francisco Correia de Almeida Fertusinhos, Rosa Maria Couceiro Pendás

**Affiliations:** 1 Unidade de Cuidados de Saúde Personalizados Chaves I-B, Chaves, Portugal; 2 Unidade de Saúde Familiar São Neutel, Chaves, Portugal

**Keywords:** Cytology, Speculum, Lubricant, Result, Pain, Citologia, Espéculo, Lubrificante, Resultado, Dor

## Abstract

**Objective** To determine if the use of lubricating gel on the speculum during the cervicovaginal cytology examination interferes with the results obtained, as well as whether it reduces reported discomfort in patients.

**Data sources** A systematic review was carried out according to the Preferred Reporting Items for Systematic Reviews and Meta-Analyses (PRISMA) recommendations, with a search in the Pubmed/Medline, Scielo, Cochrane Library, Embase databases of articles published between January 2011 and May 2022. The keywords used were *cytology* , *speculum* , *lubricant, result, and pain* .

**Selection of studies** The initial search resulted in 306 articles, of which were excluded three because they were duplicates, 257 after reading the title and abstract and 41 after reading the full text. Thus, five articles were selected for the study: four randomized clinical trials and one metanalysis.

**Data collection** The selection of articles was performed by two investigators. The 5 selected articles were read in full and submitted to a comparative analysis.

**Data synthesis** Screening through cervicovaginal cytology allows for early diagnosis and reduction of associated mortality, but the procedure can be associated with pain. A small amount of aqueous lubricating gel in the speculum can be used to reduce the discomfort associated with performing cervicovaginal cytology.

**Conclusion** The use of lubricating gel in the speculum does not seem to be associated with a change in the cytology result and reduces the discomfort associated with its insertion into the vagina.

## Resumo

**Objetivo** Determinar se o uso de gel lubrificante no espéculo durante o exame de citologia cervicovaginal interfere com os resultados obtidos e se diminui o desconforto relatado por pacientes.

**Fontes de dados** Foi realizada uma revisão sistemática segundo as recomendações do *Preferred Reporting Items for Systematic Reviews and Meta-Analyses* (PRISMA), com pesquisa nas bases de dados Pubmed/Medline, Scielo, Cochrane Library, Embase, de artigos publicados entre janeiro de 2011 e julho de 2022. Utilizaram-se as palavras-chave *citologia* , *espéculo* , *lubrificante, resultado e dor* .

**Seleção dos estudos** A pesquisa inicial resultou em 306 artigos, dos quais foram excluídos três por se encontrarem duplicados, 257 após a leitura do título e do resumo e 41 após a leitura integral. Assim, foram selecionados cinco artigos para o estudo: quatro ensaios clínicos aleatorizados e uma metanálise.

**Coleta de dados** A seleção dos artigos foi realizada por dois investigadores. Os cinco artigos selecionados foram lidos na íntegra e submetidos a uma análise comparativa.

**Síntese dos dados** O rastreio através da citologia cervicovaginal permite um diagnóstico precoce e redução da mortalidade associada, mas a sua realização pode estar associada a dor. Uma pequena quantidade de gel lubrificante aquoso pode ser utilizada no espéculo para diminuir o desconforto associado à realização da citologia cervicovaginal.

**Conclusão** A utilização de gel lubrificante não está associada a alteração do resultado da citologia e diminui o desconforto associado à sua introdução na vagina.

## Introduction

Cervicovaginal cytology (CVC) is an effective screening test for early diagnosis of cervical cancer, allowing a marked reduction in associated mortality. 1 2 3 4 According to the literature, most of the women who died from cervical cancer did not undergo cervical cytology during the previous 5 years. 3 The discomfort caused by the introduction of the speculum can decrease the rate of adherence to screening. Theoretically, the use of lubricant while introducing the speculum can minimize this discomfort and, thus, increase adherence rates for this test. 1 3 4 The Family Doctor has a key role in the prevention of disease in Primary Health Care. However, this approach is controversial, and many studies have assessed the possible effects of lubricants on cytology results. 4 5 In this sense, it was considered relevant to review the existing scientific evidence on the use of lubricating gel in the speculum during the performance of cervicovaginal cytology, as well as its influence on the results obtained and patients' reported discomfort during the test.

## Methods

A nonquantitative systematic search (evidence-based review) was performed according to the Preferred Reporting Items for Systematic Reviews and Meta-Analyses (PRISMA) recommendations. 6 Clinical guidelines, systematic reviews, meta-analyses and original studies were searched in the PUBMED databases, Cochrane Library, EMBASE, published between January 2011 and May 2022. The keywords *cytology* , *speculum* , *lubricant, result, and pain* were used. Full text studies in either Portuguese, English, or Spanish and carried out in humans were included. The following inclusion criteria were considered during study selection: 1) Population: women who underwent cervical cancer screening using the conventional technique or in liquid base; 2) Intervention: use of lubricating gel when introducing the speculum for cytology collection; 3) Comparison: use of lubricant in gel versus not using it when introducing the speculum; 4) Outcome: interference of the use of lubricant in the cytology's results and in the relief of patients' reported pain and discomfort. The following exclusion criteria were considered: duplicates, opinion articles, classic theme reviews, and articles that disagreed with this review's objective. The selection of articles for review was made by two authors who, in case of doubt, would discuss the inclusion/exclusion of the article with the third one. The agreement rate between authors in the selection of articles was 100%. All authors fully read and assessed the quality and level of evidence (LE) of the selected articles. The quality of the studies, and subsequent attribution of the level of evidence and the strength of recommendation (FR) were evaluated using the American Family Physician's Strength of Recommendation Taxonomy (SORT) scale. 7

## Results

**Fig. 1:**
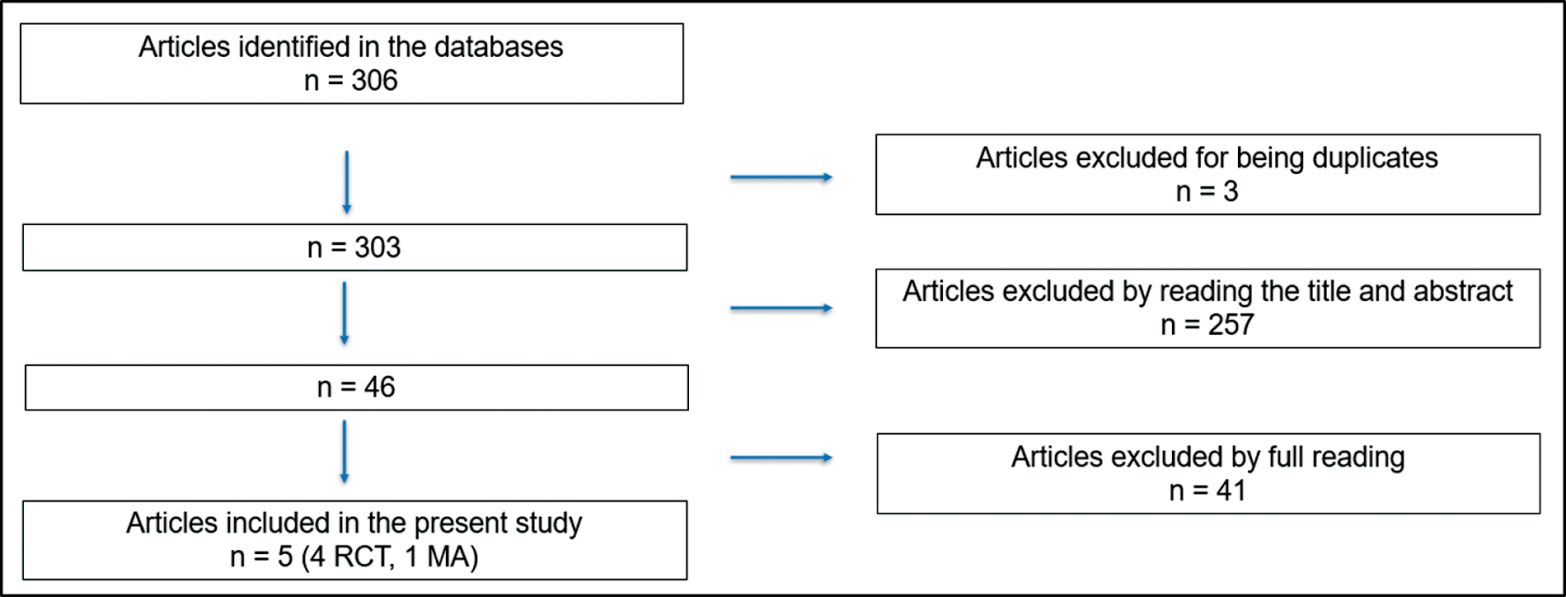
Article selection flowchart.

After searching all databases, a total of 306 articles were obtained, of which only 5 met the inclusion criteria: four randomized clinical trials (RCTs) and one metanalysis (MA). The article selection phases are specified in Figure 1 . Table 1 summarizes the characteristics of the studies selected for review and their results.

**Table 1:** Summarized description of selected articles

Ref	E	Target population and study design	Results/Conclusions	Limitations	EL
**1**	RCT	Aqueous lubricant vs. control n = 1,580. Age: 20–72 years Conventional cytology	A small quantity of aqueous lubricant diminishes reported pain by women in childbearing age and postmenopausal without altering the results of the cytology.	No evidence on other lubricant types.	1
**2**	RCT	Aqueous lubricant vs. water n = 400 Age: 23–67 years Conventional and liquid-based cytology	A small quantity of aqueous lubricant in the speculum does not affect cytology results and significantly diminishes reported pain during speculum insertion in postmenopausal women but not in childbearing age.	No evidence on other lubricant types.	1
**3**	RCT	Aqueous lubricant vs. water n = 120 Age: 18–50 years Liquid-based cytology	The use of lubricant gel was correlated with less pain in speculum insertion, without altering cytology results.	Small sample size; no postmenopausal women included; no evidence on other lubricant types.	1
**4**	RCT	Liquid petrolatum topical vs. control n = 83. Age: 15–44 years Liquid-based cytology	A small quantity of liquid petrolatum topical used as a lubricant did not alter cytology results, nor did it significantly improve reported discomfort.	Small sample size; exam performed by a wide range of physicians; few patients tested positive for epithelial lesion; no evidence on other lubricant types.	1
**5**	MA	7 studies Rates the impact of lubricant use in CVC's result and in reported pain during speculum insertion Conventional cytology	The use of aqueous lubricant does not interfere with ratio of unsatisfactory results of conventional CVC, neither does it significantly alleviate discomfort felt during procedure.	Clinical and statistic heterogeneity of included studies.	2

**Abbreviations:** CCV, cervico-vaginal cytology; E, type of study; LE, level of evidence; MA, metanalysis; Ref, bibliographic reference; RCT, randomized clinical trial.

The double-blind randomized clinical trial by Simavli et al., 1 carried out in Turkey between July 2011 and December 2012, aimed to investigate the effect of the use of aqueous lubricant on the outcome of conventional cytology, as well as on the reduction of pain or discomfort associated with vaginal introduction of the speculum. A population of 1,580 patients was evaluated, aged between 20 and 72 years, randomly divided into experimental (aqueous lubricating gel) and control (dry speculum) groups. A numerical pain scale (0–10) was used to assess pain in two phases of the procedure (introduction and opening of the speculum). They concluded that the use of a small amount of aqueous lubricant decreases the pain experienced by women, both of childbearing age and postmenopausal, without altering the quality of cytology results (unsatisfactory, premalignant lesion, or sign of inflammation) ( *p* < 0.001).

Also, in Turkey, Uygur et al., 2 carried out a double-blind randomized clinical trial, between May and September 2011, in which they studied the effect of lubrication on reported pain and on the result of conventional or liquid-based cervical cytology (unsatisfactory result, presence of abnormal cells). The study population included 400 women aged between 23 and 67 years and was randomly divided into two groups of 200 elements each: experimental (aqueous lubricating gel) and control (hot water). Each of these groups was randomly divided into two subgroups: in the first one, conventional cytology was performed; in the second one, liquid based lubricant was used during cytology. They concluded that the use of a small amount of lubricant in the speculum does not affect the interpretation of cervicovaginal cytology (conventional and liquid medium) and significantly reduces the pain caused by the introduction of the speculum in postmenopausal women ( *p* < 0.05), but not significantly in women of reproductive age.

The single-blind randomized clinical trial by Hill et al., 3 conducted in Florida between February and July 2011, studied the effectiveness of using lubricating gel in reducing pain associated with the introduction of a vaginal speculum compared to using water. It included 120 women of childbearing age, between 18 and 50 years old, with criteria for performing cytology in liquid medium. The participants were randomly divided into two groups: experimental (lubricating gel) and control (water). Pain was classified using the visual analogue scale (VAS: 0–10). It was found that the pain felt by the experimental group (with gel) was significantly lower ( *p* < 0.01). This study did not aim to evaluate the cytology results, but they were satisfactory in both groups. Hill et al. concluded that the use of a small amount of aqueous lubricating gel is associated with less pain during the introduction of the vaginal speculum, so evidence-based medicine should encourage clinicians to use aqueous lubricating gel during this procedure.

In Brazil, Nunes et al. 4 performed a double-blind randomized clinical trial between August 2012 and June 2013, with the objective of evaluating the effect of the use of lubricating gel in the speculum during cervicovaginal cytology examination. It included 83 women aged between 15 and 44 years, randomly divided into two groups: group 1 (cervicovaginal cytology was first performed with a dry speculum and later with a small amount of liquid petroleum jelly) and group 2 (both cervicovaginal cytology exams were performed with a dry speculum). There were 42 women in the first group and 43 in the second one. The exam's result and the degree of discomfort were compared between the first and second cytology, as well as between both groups. The CVC's result was evaluated by the quality of the sample (satisfactory/unsatisfactory), presence of artifacts, normal or inconclusive results, inflammation, and presence of human papilloma virus. Discomfort/pain was classified using a numerical scale from 0 to 10. There was a significantly lower discomfort when performing the second CVC in group 1 compared to group 2 ( *p* = 0.03). No significant difference was observed between the results of the first and second CVC samples in both groups. Therefore, the use of a small amount of liquid petroleum jelly as a lubricant in the introduction of the speculum did not alter the quality of the result and significantly improved the discomfort felt by participants.

Pergialiotis et al. 6 carried out a metanalysis with the objective of evaluating the impact of the use of lubricant on the CVC exam's result and reported pain when introducing the speculum. It included seven studies: five randomized clinical trials and 2 near randomized clinical trials. The authors concluded that speculum lubrication does not interfere with the rate of unsatisfactory results of conventional cytology. Since only two studies had unsatisfactory results due to lubricant use during CVC, it was not possible to draw firm conclusions. On the other hand, there was no significant relief from the discomfort felt during the examination with the use of aqueous lubricant. The studies included in this metanalysis show clinical and statistical heterogeneity.

## Discussion

In summary, the first two RCTs included both women of childbearing age and postmenopausal and concluded that the use of aqueous lubricating gel does not change the CVC's results and decreases reported pain, although in Uygur et al.'s RCTs, this conclusion only applies to postmenopausal women. 2 As for Hill et al. and Nunes et al.'s RCTs, they included only women of childbearing age and concluded that the use of lubricating gel decreases the pain felt, without altering the CVC result. 3 4 Finally, the metanalysis by Pergialiotis et al. concluded that the use of aqueous lubricant on the speculum does not interfere with the result of conventional CCV nor does it significantly alleviate the discomfort felt.

Evaluating potential limitations, it should be noted that the results cannot be generalized to any type of lubricant other than the one used in each study. Furthermore, the technique used for result collection was different between the studies. Some studies had a small sample size and did not include postmenopausal women. The included metanalysis presented heterogeneous results. The methodology of these studies did not follow a double-blind approach.

## Conclusion

The results observed here are mainly in agreement that the use of a small amount of lubricating gel in the speculum is not associated with a change in CVC exam results and, instead, can reduce the discomfort associated with its introduction into the vagina, with a grade A recommendation force.

Homogeneous studies with larger samples are recommended, to compare the effects of different types of lubricants on the CVC results and to stratify the reported pain and discomfort in pre- and postmenopausal women, in order to reinforce these results. Primary care physicians have a key role in disease prevention. Improving the comfort of women during CVC examination certainly improves adherence to cervical cancer screening, which translates to a decrease in morbidity and mortality caused by this pathology.
